# Bee Species Diversity Enhances Productivity and Stability in a Perennial Crop

**DOI:** 10.1371/journal.pone.0097307

**Published:** 2014-05-09

**Authors:** Shelley R. Rogers, David R. Tarpy, Hannah J. Burrack

**Affiliations:** Department of Entomology, North Carolina State University, Raleigh, North Carolina, United States of America; Goethe University Frankfurt, Germany

## Abstract

Wild bees provide important pollination services to agroecoystems, but the mechanisms which underlie their contribution to ecosystem functioning—and, therefore, their importance in maintaining and enhancing these services—remain unclear. We evaluated several mechanisms through which wild bees contribute to crop productivity, the stability of pollinator visitation, and the efficiency of individual pollinators in a highly bee-pollination dependent plant, highbush blueberry. We surveyed the bee community (through transect sampling and pan trapping) and measured pollination of both open- and singly-visited flowers. We found that the abundance of managed honey bees, *Apis mellifera*, and wild-bee richness were equally important in describing resulting open pollination. Wild-bee richness was a better predictor of pollination than wild-bee abundance. We also found evidence suggesting pollinator visitation (and subsequent pollination) are stabilized through the differential response of bee taxa to weather (i.e., response diversity). Variation in the individual visit efficiency of *A. mellifera* and the southeastern blueberry bee, *Habropoda laboriosa*, a wild specialist, was not associated with changes in the pollinator community. Our findings add to a growing literature that diverse pollinator communities provide more stable and productive ecosystem services.

## Introduction

Diversity is an important component of ecosystem functioning. Experimental reduction of species richness has been shown to result in productivity declines in many ecosystems [1]–[3]. Species richness (i.e., the number of different species) is positively correlated with the density and diversity of links within species interaction webs (e.g., [4]) and can stabilize ecosystem services, such as pollination, in the face of environmental variability and disturbance [5]–[7].

In agricultural systems, bee diversity may benefit pollination services in at least three ways: (1) pollinator species richness may directly improve fruit and seed set (i.e., productivity), as demonstrated in coffee [8] and pumpkin [9]; (2) diverse wild-bee communities may provide stability to pollination services, hedging against declines in managed pollinators [10], [11]; and (3) wild-bee richness may enhance the per-visit efficiency of individual pollinators within the community [12]–[13].

Positive correlations between the richness of bee species and pollination may be explained by functional complementarity, sampling effects, or both. Within an ecosystem, functional complementarity exists “when species vary in their contribution to a collective function,” such as pollination [14]. For foraging bee species, this complementarity may be temporal (time of day or season [9]) or spatial (within plants, or even flowers [9], [15]). Sampling effects, in which species-rich communities are expected to host and be dominated by more efficient taxa, may also account for the association between diversity and productivity [2]. For example, bee species differ in their pollination efficiency (defined as an individual's contribution to pollination in a single flower visit) for a particular plant species (e.g., blueberries [16]) and diverse bee communities are more likely to include the most efficient pollinators.

The ‘insurance effect’ is an often-invoked benefit of species diversity, particularly amid concerns over managed pollinator declines (e.g., [11]). Several mechanisms have been used to predict and explain enhanced stability (or decreased variability) of ecosystem services with increasing biodiversity (e.g., [11], [17]). One such stabilizing mechanism is response diversity—the differential response of organisms to environmental variability [18]. Bees exhibit a diversity of ecological and life-history traits [19], and, to a degree, these traits are predictive of a bee species' response to disturbance [20], [21]. This variability of responses suggests that diverse bee communities will be more resilient to environmental fluctuations. Evidence of response diversity related to the proportion of native flora in the landscape has been found in pollinator communities of watermelon crops [22], and response diversity with respect to variability in land use has recently been assessed—with variable results—in blueberry, cranberry, and watermelon [23].

The composition of a bee community may also influence the foraging behavior of individuals within that community [24]–[29]. In the hybrid sunflower system, in which male and female cultivars are grown in alternating rows, increases in bee abundance and species richness were positively correlated with per-visit seed set of honey bees, *Apis mellifera*, through an increased rate of interspecific encounter, avoidance, and movement of pollen-laden bees among plants [13]. Also observed in almond [12], this relationship merits examination in other types of agroecosystems, particularly in crops with different floral morphologies and cultivation practices such as highbush blueberry (*Vaccinium corymbosum*), where individual cultivars are monocropped rather than interspersed.

Theoretical work on biodiversity and ecosystem functioning has outpaced empirical field research, particularly in agroecosystems [5], [14]. In response to this deficit, we conducted an observational study of bee diversity and pollination services in highbush blueberry, a crop that is widely grown throughout the United States and Canada and hosts a diverse, well-documented bee community [30]–[35]. Though self-fertile, highbush blueberry benefits from outcrossing [36], [37] and relies on insect-pollination for agricultural production [38].

We analyzed the relationship of species richness to pollination services in highbush blueberry agroecosystems in North Carolina from each of the three perspectives described above: productivity, stability, and individual efficiency. We (1) constructed a descriptive model of pollination services to describe the relative contribution of highly-abundant managed *Apis* bees and wild bees to open-pollinated seed set; (2) tested the mechanism of response diversity in stabilizing pollinator visitation with respect to variability in foraging conditions; and (3) evaluated the per-visit efficiency of two pollinator taxa (*A. mellifera* and the southeastern blueberry bee, *Habropoda laboriosa*) along a gradient of total and heterospecific bee abundance and bee species richness that existed among sampling locations and over the course of highbush blueberry bloom. We hypothesized that there would be a positive correlation between bee diversity and all three measures of pollination services.

## Materials and Methods

### Ethics statement

All field work was conducted on privately-owned farms and with owner permission. No rare or endangered species were collected.

### Study system

We sampled the bee community and measured pollination in commercial blueberry farms in southeastern North Carolina. We selected two farms in 2010 and added a third farm in 2011. These farms were separated from one another by 10, 15, and 23 km. Each farm contained at least 40 ha of blueberries in active production. We collected pollination data from *V. corymbosum* ‘O’Neal’, a commonly grown cultivar with a long bloom period. Each farm was visited multiple times over the course of bloom from 17-Mar-2010 to 5-Apr-2010 and 14-Mar-2011 to 23-Mar-2011. We assessed pollinator visitation and subsequent pollination from one sampling location per farm in 2010, and two in 2011. Within-farm locations were separated from one another by 0.5 to 1.3 km, and were not correlated in terms of bee-community composition.

In each sampling location, we placed WatchDog A150 data loggers (Spectrum Technologies, Inc., Plainfield, IL) to record hourly temperature. Hourly solar radiation and wind speed data were obtained through a local NC Environment and Climate Observing Network station within 50 km of farms. We quantified the percent bloom during each visit by counting all flowers (in either bud, bloom, or petal-fall stages) on a single branch from four contiguous plants per sampling location. These four plants were located in the center of a cultivar block, between two rows where bee community observations and blueberry pollination trials were conducted. We calculated the proportion of flowers that had developed beyond the bud stage. Based on this proportion, we categorized the phenological stage of a planting as either early- (≤0.33), middle- (0.33< p ≤0.66), or late-bloom (>0.66). We sampled each location at least once per bloom stage, with the exception of one farm during the first year of the study, conducting a total of 24 location visits.

### Bee community

To assess the composition of the pollinator community, we counted all bees observed foraging along two transects per sampling location. In 2010, we traversed each 60-m transect in two minutes, while in 2011, we sampled 30-m transects for the same length of time. We, therefore, doubled 2011 bee counts to standardize for sampling effort and ensured that this correction factor did not bias observations in either direction. We conducted transect walks at 900, 1100, 1300, and 1500 h for a total of eight transect samples per location visit. We counted all bees actively foraging at flowers—including those engaged in nectar robbing [39]—and classified these into five groups that could be distinguished on the wing: *Apis mellifera*, *Bombus* spp., *Habropoda laboriosa, ‘*small native’ bees, and *Xylocopa virginica*; hereafter, bee groups will be referenced by genus name only. Based on transect counts, we calculated bee abundance and richness (number of species groups, described above) for location visit.

We employed pan trapping to complement our transect sampling and to enable genus- and species-level identification of bees. We constructed pan traps from plastic, 96-ml soufflé cups painted either white, fluorescent yellow, or fluorescent blue, and filled halfway with soapy water [40]. Using Velcro, we attached pans atop 1.2-m step-in posts to elevate traps into the plant canopy (see [41]). In 2010, we placed two trap lines with 10 traps per line in the same cultivar block in which timed bee observations were conducted. Equal numbers of each color pan trap were randomly ordered and spaced 3 meters apart within rows [42]. In 2011, we increased pan trap sampling in each cultivar block to three trap lines with 12 pan traps per line. We adjusted 2010 pan trap captures by a factor of 1.8 to account for differences in sampling effort. We separated trap lines from each other by three rows of plants and from bee observation transects by one row of plants. Row spacing ranged from 3.0 to 3.6 m apart. We established traps at 800 h and collected them at 1600 h during each visit.

We identified pan-trapped specimens to genus and species level, as practical. We used both published and online guides—Michener et al. [43], Mitchell [44], [45], and the Apoidea section of www.discoverlife.org-and the North Carolina State University Insect Museum reference collection to identify bees. Voucher specimens have been deposited in this same museum.

### Blueberry pollination

We sampled fruits resulting from both open-pollinated flowers and flowers visited by a single bee. To determine per-visit pollinator efficiency, we placed No-see-um mesh (Denver Fabrics, Denver, CO) cages on branches without open flowers (which were removed as necessary). Depending on the density of unopened flowers, we caged up to two branches per plant on four to eight plants per sampling location. During bloom, we returned, removed cages and observed virgin flowers for visitation by either *Apis* or *Habropoda*. Though other bees were common at flowers, *Apis* and *Habropoda* were particularly abundant across farms and years, and represent poly- and oligo-lectic pollinators, respectively, present in blueberry culture throughout the southeast [31]. Once visited by a bee, we marked a flower by tying embroidery thread around the pedicel and distinguished flowers visited by *Apis* and *Habropoda* with different thread colors. We then immediately placed a smaller cage around the visited flower(s) to prevent subsequent visitation. We left flowers (five per plant) that were in a similar stage of bloom uncaged for the open pollination treatment. Each caged branch was labeled with the observation date, and not observed on subsequent visits to the same sampling location. Pollination treatments were established on the same day that bee observations and pan-trapping took place.

We collected fruit samples approximately 50 days following each location visit. We placed berries in cold storage until they could be dissected to count seeds. Blueberries produce both viable and non-viable seeds [36]. We counted apparently-viable seeds to quantify seed set per fruit. In 2010, we counted only ‘large’ seeds as viable; in 2011, we counted seeds >1.3 mm in length as viable (for methods, see [16]). Blueberry seed set is positively correlated with both fruit weight and volume [16], but it is a more direct measure of pollination and less sensitive to cultural practices such as irrigation, pruning, weed management, and plant spacing.

### Statistical analysis

We calculated the number of bees from each group counted in a day's worth of sampling effort (two transects, evaluated four times) per location. We then counted the number of different species groups (species richness) present in transects from the same sampling period. We tested for correlation between transect and pan trap observations for each bee group using a multivariate analysis of variance in SAS Proc GLM (for all analyses: SAS version 9.2, SAS Institute, Cary, NC, USA). Our response variables were transect and pan trap counts; farm and year were included as random effects. We also tested for correlation within and between species group densities (both *Apis* and wild bee groups) and community measures in Proc GLM.

We analyzed pollination through a generalized-linear mixed model (SAS Proc GLIMMIX). Our response variable was open-pollinated seed set per fruit. Due to correlation among the densities of wild-bee groups (*Bombus*, *Habropoda*, ‘small native’, and *Xylocopa*), we combined their counts into one ‘wild bee’ group. Our predictor variables were *Apis* abundance, wild-bee abundance and richness, and year. Though correlated, we included both wild-bee abundance and richness in the model to determine which was more informative. *Apis* and wild-bee abundance were logarithmically related to seed set, thus, we log-transformed [log(x+1)] both variables in our full model. Because of differences in the 2010 and 2011 seed count methods, we included year as a fixed effect for all models in which seed set was the response variable. *Apis* abundance was positively correlated with temperature and solar radiation and negatively correlated with wind speed (see [16]). Thus, these weather variables were not included as covariates in the model. We expect that the direct effect of these variables on pollination is negligible compared with their indirect effect on pollination via the bee community. Farm and plant (nested within year, farm, and visit) were treated as random effects. To account for differences in sample size (berries per sampling period), we used the Satterwhaite method to approximate degrees of freedom. Because a *r^2^* statistic cannot be obtained through maximum-likelihood estimation methods, we calculated Efron's pseudo *r^2^* [1 – (sum of variance components)/(total variance)] to estimate the descriptive value of our model.

To test the hypothesis of response diversity of pollinator taxa to variation in weather, we evaluated the interaction between taxa and weather condition in a model describing pollinator visitation (number of bees per group counted in transect walks per day of sampling effort), a method first developed and employed by Winfree and Kremen [22]. Bee counts were square-root transformed to normalize the distribution of residuals. Pollinator taxa were *Apis*, *Bombus*, *Habropoda*, ‘small native’, and *Xylocopa*. Daily weather variables (temperature, solar radiation, and wind speed) were calculated as the mean of hourly weather collected between 900 and 1700 hours (when foragers are active). Daily weather conditions were classified as ‘optimal’ if temperature ≥19°C, solar radiation ≥510 W m^−2^, and wind speed ≤11 km h^−1^. Daily weather that did not satisfy all of these conditions was classified as ‘inclement’. There was a minimum difference of 1°C, 50 W m^−2^, and 2 km h^−1^ between individual measurements that were described as ‘optimal’ or ‘inclement’. We conducted the analysis in SAS Proc GLIMMIX to allow for incorporation of random effects: farm by year, and visit (nested within farm and year).

We compared the forager abundance of each pollinator group in ‘inclement’ and ‘optimal’ weather conditions using a generalized-linear mixed model (SAS Proc GLIMMIX) with bee abundance as the dependent variable, and weather condition as the fixed effect. Farm and year were included as random effects. We then compared pollination (seed set) in both weather conditions. For this analysis, our fixed effects included weather and year. We treated farm and plant (nested within year, farm, and visit) as random effects.

We analyzed the per-visit efficiency of *Apis* and *Habropoda* with respect to both total and heterospecific bee abundance, and total bee species richness. The proportion of viable seeds (based on the maximum observed seed set of 93) was arcsin-square root transformed for normality. Solar radiation, which we suspected of affecting the physical extraction and dispersal of pollen, and year were included as fixed effects in all models. We retained all variables for which *p*<0.25 (see [46]). We included farm and plant (nested within year, farm, and visit) as random effects in SAS Proc GLIMMIX and used the Satterwhaite approximation of degrees of freedom. From these analyses we present back-transformed estimates of per-visit seed set ± 95% CI.

### Analysis of economic impact

From the generalized-linear mixed model of pollinator contribution to seed set, we calculated the economic value associated with the presence of each additional wild-bee group during bloom. We first estimated the slope of viable seeds to berry mass from all open-pollinated fruits, using the same analysis structure described above for our evaluation of pollination services. With this value, the 2011 grower price for berries (by weight) in NC [47], and the model estimate of seed set per wild bee group richness, we calculated the change in the dollar value of a single berry (based on a change in berry mass in response to wild bee group richness). Using berry mass and yield data (berries per hectare) [47], [48], we then estimated the number of berries per hectare and, from this, the change in economic value per hectare. We determined the economic value per wild bee group richness for all highbush blueberry grown in the area (total acreage >1800 ha). We performed this computation as above, but used cultivar-specific values for berry mass [48] in the calculation of berries per hectare, and weighted the change in economic value per hectare by the land area covered by each cultivar (see table S1 in File S1 for calculations).

## Results

### Bee community

We observed a total of 2,177 bees in transect sampling and captured 219 bees in pan traps in 24 location visits, comprising 192 transect samples, over the course of highbush blueberry bloom in 2010 and 2011 (table 1). From pan-trapped specimens, we identified five families (Andrenidae, Apidae, Colletidae, Halictidae, and Megachilidae) and 12 genera of bees. One species, *Andrena bradleyi*, was highly abundant at all stages of bloom, constituting 33 to 61% of all pan-trapped ‘small native’ bees. Pan trap captures were not correlated with transect counts for *Apis* (*r* = 0.14, *p* = 0.53), *Habropoda* (*r* = 0.13, *p* = 0.57), or ‘small native’ bees (*r* = 0.11, *p* = 0.64), and no *Xylocopa* were collected in pan traps. Pan trap and transect observations were correlated for *Bombus* (*r* = 0.47, *p* = 0.03). For the subsequent analyses, transect data only were used for measures of bee abundance and richness.

**Table 1:** Bee species observed in transects and pan traps during blueberry bloom.

bloom stage	early		middle		late	
sampling effort (days)	n = 9		n = 7		n = 8	
method	transect	pans	transect	pans	transect	pans
Apis mellifera*	**227**	**65**	**504**	**34**	**1036**	**28**
*Bombus* spp.*	**1**	**1**	**8**	**2**	**11**	**1**
*B. bimaculatus*		1		2		0
*B. impatiens*		0		0		1
Habropoda laboriosa*	**90**	**2**	**116**	**2**	**52**	**2**
‘small native' bees*	**3**	**31**	**27**	**33**	**40**	**18**
*Agapostemon splendens*		0		1		1
Andrena bradleyi*		19		20		6
*Andrena* spp. *		6		8		6
Augochlora pura*		1		1		1
*Ceratina* spp.		1		0		0
*Colletes* spp.		1		0		1
*Halictus rubicundus*		0		1		0
*Lasioglossum* (*Dialictus*) spp.		2		0		3
*Lasioglossum* (*Evylaeus*) spp.		0		1		0
*Nomada* spp.		1		1		0
Xylocopa virginica*	**7**	**0**	**17**	**0**	**38**	**0**
**total bees**	**328**	**99**	**672**	**71**	**1177**	**49**

Transect and pan trap counts represent the total number of bees observed at locations on multiple visits (indicated by sampling effort) in 2010 and 2011. Totals for each bee group are provided in bold above the individual counts of any species comprising that group. Bees observed at all sampling locations (in either transects or traps) are indicated with an asterisk (*).

### Productivity

In biodiversity-productivity studies, species richness and total abundance are often positively confounded, making it difficult to distinguish their contribution to ecosystem services [6]. Though we did not control pollinator density at our sampling locations, bee abundance and species richness (total or wild bee) were not significantly correlated in our system (total richness, *r* = 0.32, *p* = 0.13; wild bee richness, *r* = 0.25, *p* = 0.24), enabling us to consider their contributions separately. We suspect the independence of these factors is due to the importation of managed *Apis*, as bee abundance was highly correlated with *Apis* abundance (*r* = 0.96, *p*<0.0001), while spatial and temporal factors may drive species richness. Wild-bee abundance and richness were correlated (*r* = 0.77, *p*<0.0001), but neither measure was related to *Apis* abundance (wild-bee abundance, *r* = 0.12, *p* = 0.57; wild-bee richness, *r* = 0.04, *p* = 0.86).

**Figure 1:**
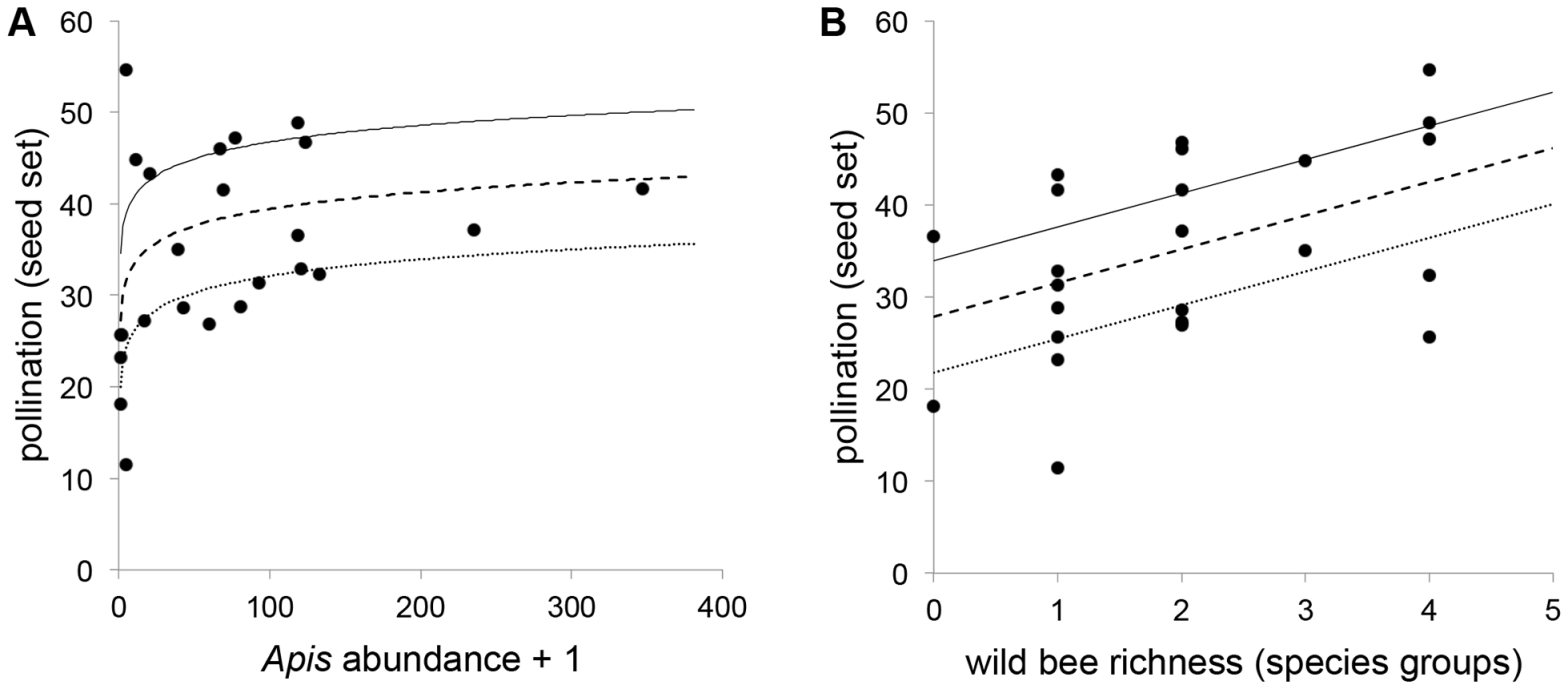
Relationship of pollination to *Apis* abundance (*a*) and wild-bee richness (*b*) in highbush blueberry. Each point represents the mean seed set per location visit. Trend lines are based on model estimates described in table 2 (using mean of year estimates) at different levels of *Apis* and wild-bee richness. For (*a*), logarithmic fits represent wild-bee richness of 0 (dotted line), 2 (dashed line), and 4 (solid line). For (*b*), linear fits represent *Apis* abundance of 2 (dotted line), 20 (dashed line), and 200 (solid line).

Both *Apis* abundance (figure 1*a*) and wild-bee richness (figure 1*b*) were significant factors describing pollination (seed set) in blueberry (table 2; n = 550, pseudo *r^2^* = 0.58). Wild-bee abundance (log-transformed) was less informative than richness (*F  = *0.46 and 1.45, respectively) and was removed from the final model. However, if richness is excluded from the model, wild-bee abundance is significantly correlated with pollination (est ± SE: 4.06±1.24, *F*  = 10.80, *p* = 0.0014). Seed set was significantly higher in 2010 than 2011; we suspect this reflects the difference in our seed count methods, rather than biological changes [see 16]. Location and visit-specific bee and pollination data are included in Tables S2 and S3 in File S1.

**Table 2:** Descriptive model of pollination services (quantified as open-pollinated seed set) provided by *Apis* and wild bees in highbush blueberry.

fixed effects	estimate ± SE	*F*	*p-*value
Intercept	16.83±4.29	15.37	0.0012
*Apis* abundance*	2.64±0.84	9.95	0.0022
wild-bee richness	3.66±1.06	11.91	0.0007
year †	−6.24±2.99	4.34	0.0391

Values reported are result of generalized-linear mixed models analysis with Type III test of fixed effects. *Data were log-transformed. †Estimate represents difference of 2011 from 2010.

### Response diversity

In 2010, weather conditions were ‘optimal’ on two days and ‘inclement’ on four days. In 2011, weather conditions were ‘optimal’ on three days and ‘inclement’ on six days. In both years, ‘inclement’ weather conditions were experienced during each bloom stage. The mean weather conditions (± SE) were 23±1°C, 597±30 W m^−2^, and 8±1 km h^−1^ on ‘optimal’ days and 19±1°C, 511±30 W m^−2^, and 13±1 km h^−1^ on ‘inclement’ days. As observed, mean weather data for ‘inclement’ days could exceed the ‘optimal’ condition for any individual criteria because weather conditions had to fail to meet only one of the three criteria to be classified as ‘inclement’.

**Figure 2:**
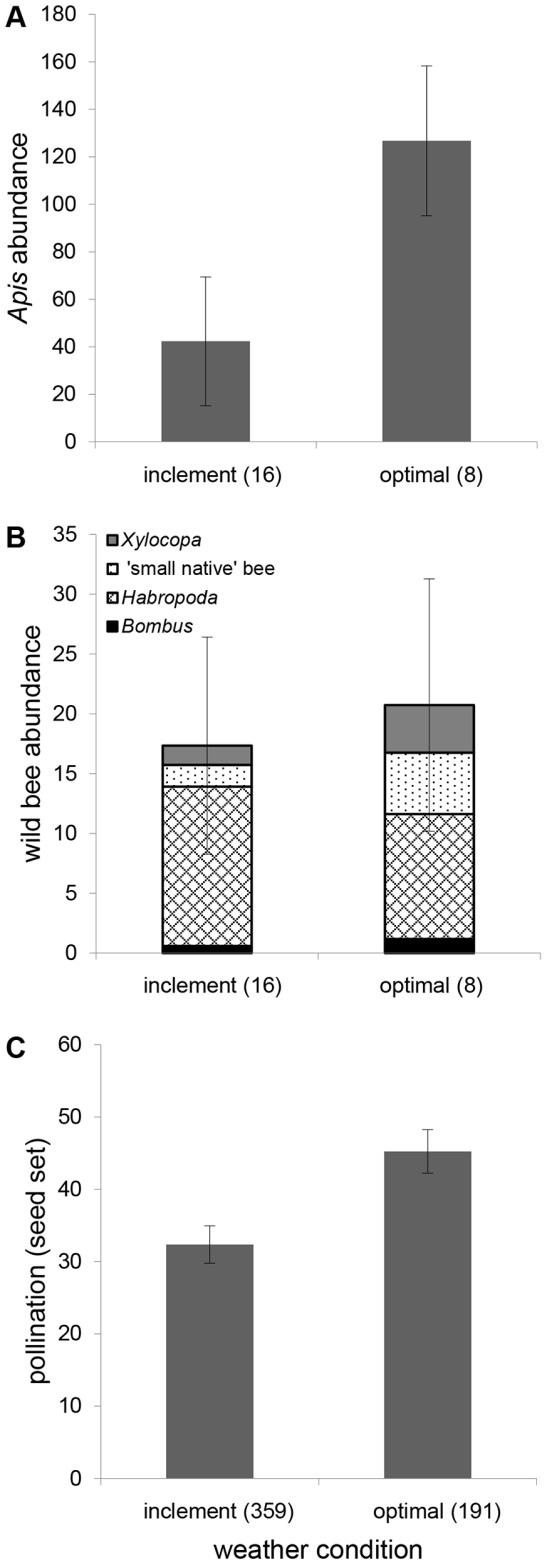
Forager abundance and pollination during ‘inclement’ and ‘optimal’ weather conditions in blueberry fields. Mean estimates with SE bars shown for *Apis* abundance (*a*), wild-bee abundance (*b*), and open-pollinated seed set (*c*). Replicates, included in parentheses, are location visits (*a, b*) and number of berries (*c*).

The bee pollinators present in our system exhibited response diversity with respect to weather, indicated by the significant interaction between taxa and weather condition (table 3). Whereas *Apis* were three times less abundant in inclement than optimal weather conditions (*p* = 0.009, figure 2*a*), overall wild bee density did not differ (*p* = 0.71, figure 2*b*). Within wild bee groups, however, ‘small natives’ were significantly less abundant in inclement weather (*p* = 0.05, figure 2*b*) while *Bombus*, *Habropoda*, and *Xylocopa* abundance remained stable (*p* = 0.4, 0.7, 0.3, respesctively, figure 2*b*). On average, blueberry experienced reduced pollination as indicated by seed set (−12.9 seeds fruit^−1^) during periods of inclement weather (*p*<0.0001, figure 2*c*).

**Table 3:** Analysis of response diversity of blueberry pollinators to daily weather conditions.

response variable			
number of bees observed in transect walks			
source of variation	df	*F*	*p-*value
taxa^a^	4, 98	41.36	<0.0001
weather^b^	1, 13	4.81	0.047
taxa × weather	4, 98	6.42	0.0001

Bee counts were square-root transformed. Values reported are result of generalized-linear mixed model with Type III test of fixed effects.

a Bees were classified into five species groups (*Apis*, *Bombus*, *Habropoda*, ‘small native’, and *Xylocopa*).

b Daily weather conditions were classified as ‘inclement’ or ‘optimal.’

### Community effects on per-visit efficiency

The per-visit efficiency of *Apis* (n = 238) was not affected by wild (*F  = *0.03, *p* = 0.85) or total bee abundance (*F  = *0.54, *p* = 0.47), or richness (*F  = *0.07, *p* = 0.79). *Habropoda* per-visit efficiency (n = 72) was not correlated with the abundance of heterospecific bees (*F  = *1.70, *p* = 0.21) but trended negatively with total bee abundance (−0.03±0.01 seeds visit^−1^, *F  = *3.26, *p* = 0.08) and richness (−0.32±0.23, *F  = *3.70, *p* = 0.06). In the reduced model, both *Apis* and *Habropoda* per-visit efficiency trended positively with solar radiation (*Apis*, 0.0009±0.0002 seeds per 100 W m^−2^, *F  = *3.58, *p* = 0.06; *Habropoda*, 0.005±0.001 seeds per 100 W m^−2^, *F  = *3.57, *p* = 0.07) but were not affected by year (*F  = *0.16, *p* = 0.69, and *F  = *0.66, *p* = 0.43, respectively).

### Economic impact of wild bee richness

For each additional species group present during bloom (richness + 1), fruits produced an average of 3.66 more viable seeds (Table 2). This change in seed set translates to an increase in economic value of $757 ha^−1^ for ‘O’Neal’ (the cultivar we surveyed) and $1,424,000 for all highbush blueberry grown in North Carolina.

## Discussion

Though they may be intensively managed, farms function as ecosystems and are often situated in or near unmanaged landscapes that provide additional ecosystem services [49], [50] and may influence pollinator diversity [23]. Diversity is an important component of how these ecosystems function, yet the mechanisms are often poorly understood in agricultural settings [5]. In highbush blueberry, we find that wild-bee diversity improves ecosystem functioning by enhancing pollination services (quantified as seed set) and the stability of pollinator visitation in variable climatic conditions.

This study is the first to document the pollinator community of highbush blueberry agroecosystems in North Carolina. In addition to managed *Apis*, wild bees were common at blueberry flowers, accounting for nearly a fifth of all visits. *Habropoda laboriosa* and *Andrena bradleyi*, two *Vaccinium* oligoleges that sonicate flowers to release pollen tetrads, were abundant at all sampling locations. While *H. laboriosa* has been enthusiastically studied in southeastern blueberry systems [51]–[53], less is known about *A. bradleyi*, which we frequently observed foraging in transects. We did not count *A. bradleyi* separately from other ‘small natives,’ because we did not learn to distinguish this species until into our second year of sampling. *A. bradleyi* has been documented in *Vaccinium* crops, including cranberry, along the eastern US coast (Massachusetts [54], Maine [55], and Nova Scotia [56]) but not in more interior (Michigan [35]) and western (British Columbia [34]) farms. Recent work suggests that *A. bradleyi* is positively associated with agricultural habitat and, therefore, may play a significant role in the stability of these systems [23].

Pan traps have many benefits [57], yet they may poorly sample the bee community when in competition with abundant floral resources [58] and under-represent large bees such as *Apis* [57] and *Bombus* [59]. Our findings in blueberry suggest that pan traps are indeed ineffective at documenting the relative abundance of bees visiting a mass-flowering crop. Pan-trap captures were not correlated with direct observations for *Apis*, *Habropoda*, ‘small native’ bees, or *Xylocopa*. To our surprise, this was not the case for *Bombus* spp., though only a total of five bees were caught in two years of sampling. In the absence of direct observations or sweep netting, pan traps may lead to false conclusions about the relative importance of different bee species in agricultural landscapes.

In our model of pollination services, *Apis* and wild bees were equally descriptive of seed set. Pollination increased logarithmically with *Apis* density, indicating relative saturation at higher densities. ‘Wild bees’ included *Bombus*, *Habropoda*, ‘small natives’, and *Xylocopa*, the densities of which were correlated and, therefore, could not be considered separately, although these taxa differ in foraging strategy. Because wild-bee abundance and richness were highly correlated, we could not consider their contribution to pollination independently. However, wild bee richness was more informative of blueberry pollination. This is suggestive of two mechanisms: a sampling effect (species-rich bee communities may be more likely to host more efficient blueberry pollinators such as *Bombus* and ‘small native’ bees [16]) and functional complementarity (a species-rich community may host more functionally-diverse groups that visit plants in complementary ways [9]).

The positive, linear relationship between seed set and wild-bee richness in an agricultural crop enabled us to further consider possible quantification of pollination services. Our general model simplifies the variation in efficiency for each species, as the number of seeds resulting from single visits by blueberry-pollinating wild bees varies between groups [16]. However, our model clearly demonstrates benefit from the presence of additional species groups, and on average that benefit translates to an increase of 3.66 seeds berry^−1^group^−1^. Scaled over the state where our observations were made, this represents a meaningful increase in crop value (2% of total annual value) for each new wild-bee group. Though we only observed a linear relationship between pollination and bee-group richness for the range of species groups in our study, we expect this relationship is ultimately asymptotic. Because the farms we surveyed are representative (in size, management, and geography) of those comprising the bulk of production in North Carolina, our economic analysis provides a reasonable estimate of the value of species richness in highbush blueberry for that state. We suspect the relationship between species richness and production, though qualitatively consistent, is quantitatively different for other blueberry systems (e.g., in Michigan) where farm size, management, and environmental variables differ from our own, and, thus, we caution against the over extrapolation of our economic findings.

In eastern North Carolina, highbush blueberry blooms for several weeks in early spring and is subject to variable weather conditions during this time. As weather influences the foraging behavior or bees (e.g., [60]), it is an essential component of pollinator community dynamics in these agroecosystems. In Michigan blueberry systems, *Apis* were the dominant flower visitors during ‘good’ weather, while *Bombus* were dominant in ‘poor’ weather [20]. Similarly, our analysis of response diversity shows that blueberry pollinators respond differently to changes in weather, with the number of *Apis* foragers dramatically reduced in inclement weather conditions. Wild bees were less affected by changes in weather, with *Habropoda* being slightly more abundant in inclement weather. *H. laboriosa*'s ability to forage in variable weather may have been either a factor in or product of its evolutionary association with *Vaccinium*. This observed resilience in non-*Apis* pollinators may reduce, but not totally offset, the temporal variability of pollination services. Moreover, response diversity may act as a form of functional complementarity, enhancing ecosystem productivity, in addition to stability [14]. Blueberry pollinators have also been shown to exhibit response diversity to surrounding land cover [23].

We found that *Apis* and *Habropoda* per-visit efficiencies were not significantly correlated with total- or heterospecific-bee abundance, or bee-species richness. Taken together with those of Greenleaf and Kremen [13] and Brittain et al. [12], our findings suggest that community effects on per-visit efficiency are system dependent. Almond, hybrid sunflower, and highbush blueberry represent three very different cropping systems. In the former two crops, male and female cultivars are alternated between rows. In highbush blueberry, a single, clonally-propagated cultivar is often planted over a large area (several hectares), so that the movement of bees between plants may not improve the transfer of xenogamous pollen [61]. Hence cultural practices may constrain variation in per-visit efficiency in this system. Our understanding of the relationship between bee community and pollinator efficiency would benefit from future work in other pollinator-dependent systems (such as cucurbits and apples) with diverse flower morphologies and cropping practices. With respect to fruit set, rather than per visit efficiency, large-scale meta analysis also demonstrates that contributions by wild bees and honey bees are not dependent upon one another [62].

We have demonstrated two important ways that wild bees enhance pollination success: increased productivity and temporal stability. These findings add to a growing body of research showing that wild-bee communities provide important pollination services to agroecosystems [8], [9], [50].

## Supporting Information

File S1 Contains Table S1, calculation of economic impact of species group richness for highbush blueberry. Table S2, mean (±SE) pollinator visitation and seed set per location. Table S3, mean (±SE) pollinator visitation and seed set per bloom stage.
(DOC)
