# Supplementary material for: Bee Species Diversity Enhances Productivity and Stability in a Perennial Crop
Source: PLoS One. 2014 May 9;9(5):e97307. doi: 10.1371/journal.pone.0097307 (PMC4016306; doi:10.1371/journal.pone.0097307)
Supplement: File S1 — Contains Table S1, calculation of economic impact of species group richness for highbush blueberry. Table S2, mean (±SE) pollinator visitation and seed set per location. Table S3, mean (±SE) pollinator visitation and seed set per bloom stage. (DOC) [file pone.0097307.s001.doc]

**Table S1. Calculation of economic impact of species group richness for highbush blueberry.**

| **cultivar** | **cultivation *^c^*** | **berry yield *^d^*** | **∆ economic yield*^e^*** | **∆ economic value *^f^*** |
| --- | --- | --- | --- | --- |
| (unit) | (ha) | (10^6^ fruits ha^-1^) | ($ ha^-1^) | ($1000s) |
| ‘O’Neal’ | 300 | 4.00 | 757 | 227 |
| ‘Legacy’ | 400 | 4.70 | 889 | 356 |
| ‘Star’ | 300 | 3.33 | 631 | 189 |
| ‘Duke’ | 250 | 3.66 | 693 | 173 |
| ‘Croatan’ | 200 | 4.35 | 823 | 165 |
| ‘Reveille’ | 150 | 5.88 | 1,113 | 167 |
| ‘Rebel’ | 50 | 3.61 | 683 | 34 |
| Other *^a^* | 200 | 2.99 | 565 | 113 |
| **total** | **1,850** | **4.07*^g^*** | **769*^g^*** | **1,424** |

*^a^* Other highbush cultivars (n=13), each with less than 50 ha under cultivation. *^b^* The mean berry mass from cultivars (‘New Hanover,’ ‘Emerald,’ ‘San Joaquin,’ and ‘Jewel’) for which data is available. *^c^* Land area estimates for North Carolina provided by Bill Cline (*pers comm*). *^d^* The per-cultivar berry yield is the product of the average yield (by mass) for NC in 2011 (7000 kg ha^-1^; USDA ERS 2012) and the inverse berry mass; berry mass values were obtained from Mainland [48]. *^e^* The change in economic yield ($ ha^-1^) for each additional wild bee group present is the product of four values: (1) the change in seed set per increase in wild bee richness (3.66 seeds berry^-1^; see Table 2); (2) the slope of all viable seeds to berry mass, calculated from 953 open-pollinated fruits (0.0128 ± 0.0003 g seed^-1^, *p* < 0.0001); (3) the 2011 grower price for berries in NC (weighted by utilization, $4.04 kg^-1^; USDA ERS 2012); and (4) the per-cultivar yield (berries ha^-1^) from the previous column. *^f^* The change in economic value for blueberry production in North Carolina is the product of the per-cultivar land area and change in economic yield ($ ha^-1^) from the previous column. *^g^* Values represent means, not sums.

**Table S2. Mean (±SE) pollinator visitation and seed set per location.**

| **year** | **farm** | **location** | ***Apis*** | **wild bees** | **wild bee richness***^a^* | **seed set** |
| --- | --- | --- | --- | --- | --- | --- |
| 2010 | A | 1 | 121 ± 3 | 6 ± 6 | 1 ± 1 | 42 ± 5 |
|  | B | 1 | 16 ± 14 | 42 ± 20 | 3 ± 1 | 33 ± 7 |
| 2011 | A | 1 | 105 ± 69 | 3 ± 2 | 1 ± 1 | 28 ± 6 |
|  |  | 2 | 165 ± 97 | 15 ± 10 | 3 ± 1 | 34 ± 4 |
|  | B | 1 | 59 ± 8 | 9 ± 4 | 2 ± 0 | 39 ± 5 |
|  |  | 2 | 23 ± 8 | 12 ± 5 | 2 ± 1 | 41 ± 3 |
|  | C | 1 | 56 ± 28 | 9 ± 4 | 2 ± 1 | 35 ± 6 |
|  |  | 2 | 81 ± 38 | 30 ± 19 | 2 ± 1 | 31 ± 11 |

Transect observations (two transects, observed four times per visit) and seed set are averaged over consecutive location visits.

**Table S3. Mean (±SE) pollinator visitation and seed set per bloom stage.**

| **farm** | **bloom stage** | ***Apis*** | **wild bees** | **wild bee richness** | **seed set** |
| --- | --- | --- | --- | --- | --- |
| A | Early | 45 ± 37 | 1 ± 1 | 1 ± 1 | 27 ± 5 |
|  | Middle | 106 ± 26 | 18 ± 16 | 3 ± 2 | 31 ± 2 |
|  | Late | 234 ± 64 | 8 ± 2 | 2 ± 0 | 42 ± 3 |
| B | Early | 1 ± 1 | 22 ± 19 | 2 ± 1 | 21 ± 3 |
|  | Middle | 72 ± 35 | 33 ± 21 | 2 ± 1 | 40 ± 8 |
|  | Late | 84 ± 18 | 32 ± 17 | 3 ± 1 | 41 ± 7 |
| C | Early | 44 ± 24 | 5 ± 3 | 1 ± 0 | 42 ± 1 |
|  | Middle | 38 ± 28 | 16 ± 6 | 3 ± 1 | 45 ± 1 |
|  | Late | 40 ± 2 | 10 ± 4 | 3 ± 1 | 32 ± 3 |

Transect observations (two transects, observed four times per visit) and seed set are averaged over year and within-farm location.
